# Low Levels of Vitamin D Promote Memory B Cells in Lupus

**DOI:** 10.3390/nu12020291

**Published:** 2020-01-22

**Authors:** Erin A. Yamamoto, Jane K. Nguyen, Jessica Liu, Emma Keller, Nicole Campbell, Cun-Jin Zhang, Howard R. Smith, Xiaoxia Li, Trine N Jørgensen

**Affiliations:** 1 Cleveland Clinic Lerner College of Medicine of Case Western Reserve University, Cleveland, OH 44195, USA; 2 Lerner Research Institute, Department of Inflammation and Immunity, Lerner Research Institute, Cleveland Clinic, Cleveland, OH 44195, USA; 3 Robert J. Tomsich Pathology and Laboratory Medicine Institute, Cleveland Clinic, Cleveland, OH 44195, USA; 4 Department of Rheumatologic and Immunologic Disease, Cleveland Clinic, Cleveland, OH 44195, USA

**Keywords:** Systemic Lupus Erythematosus, vitamin D3, memory B cells, Th17 cells, Act1

## Abstract

*Background*: Vitamin D deficiency is a known risk factor for Systemic Lupus Erythematosus (SLE), yet clinical trials have not demonstrated efficacy and few studies have utilized lupus models to understand the mechanism underlying this relationship. The *Act1-/-* mouse is a spontaneous model of lupus and Sjögren’s syndrome, characterized by increased Th17 cells and peripheral B cell expansion. Vitamin D3 has anti-inflammatory properties, reduces Th17 cells and impairs B cell differentiation/activation. Therefore, we assessed how varying amounts of vitamin D3 affected lupus-like disease in the *Act1-/-* mouse. *Methods*: *Act1-/-* mice were fed either low/restricted (0 IU/kg), normal (2 IU/kg), or high/supplemented (10 IU/kg) vitamin D3 chow for 9 weeks, after which lupus-like features were analyzed. *Results*: While we found no differences in Th17 cells between vitamin D3 groups, vitamin D3 restriction specifically promoted memory B cell development, accompanied by elevated levels of serum IgM, IgG1, IgG3, and anti-dsDNA IgG. A similar significant negative association between serum vitamin D and memory B cells was confirmed in a cohort of SLE patients. *Conclusion*: Low levels of vitamin D3 are associated with elevated levels of memory B cells in an animal model of lupus and well-controlled SLE patients.

## 1. Introduction

Systemic Lupus Erythematosus (SLE) is an autoimmune disease predominantly affecting women of child-bearing age at a rate of 20 to 150 cases per 100,000 population [1]. Genetic, environmental, and hormonal factors all contribute to SLE pathogenesis, and no single factor elicits disease if encountered alone. Different combinations of risk factors lead to variable and unique patient presentations, including photosensitivity, rash, arthritis, cytopenias, serositis, nephritis, fatigue, and psychosis [1]. While the pathophysiology of SLE is incompletely understood, it is characterized by aberrant T and B cell activity, elevated autoantibody titers, and subsequent organ damage. The characteristic presence of autoantibodies (e.g., anti-dsDNA IgG, anti-chromatin IgG, anti-Sm IgG), support a pathologic role for B cells in SLE. Additionally, autoantibody titers have been shown to increase with disease activity, and newer immunotherapies (e.g., rituximab, belimumab) that target B cells have demonstrated some benefit [2,3].

Vitamin D deficiency is a common laboratory finding in many autoimmune diseases including SLE [4]. Vitamin D deficiency is inversely correlated with lupus disease activity, and mutations in the vitamin D receptor (VDR) have been identified in SLE populations [5]. A couple of studies have investigated the relationship between vitamin D3 supplementation and SLE in animal models, demonstrating an overall protective effect ([6,7,8] and reviewed in [9]). Although evidence from these studies suggests that vitamin D3 plays a protective role in lupus, clinical supplementation studies in SLE patients have demonstrated inconclusive therapeutic benefits [10,11,12,13]. Thus, whether supplementation or deficiency of vitamin D3 contributes to disease prevention or susceptibility, respectively, and the mechanism(s) by which such regulation would occur remains unknown.

Immune cells (monocytes, dendritic cells, macrophages, activated lymphocytes) express VDR and 1α-hydroxylase, the key enzyme that produces the biologically active form of vitamin D3 (1,25(OH)_2_D3) from its precursor 25(OH)D3. Vitamin D3 has immune-modulating properties, such as an ability to inhibit Th1 and Th17 differentiation, while promoting Treg development [14,15,16]. Additionally, vitamin D3 has been shown to inhibit B cell proliferation [17,18,19], and differentiation into memory B cells and antibody-secreting plasma cells [20,21,22]. There is also a reduction in immunoglobulin production [21,22], suggesting a role for vitamin D3 in regulating differentiation and/or activity of downstream memory B cells or plasma cells. The specific role of vitamin D3 in B cell differentiation and autoantibody secretion in animal models of SLE, however, has not been determined.

The Act1 (TRAF3IP2 or CIKS) knockout mouse is a model for SLE and Sjogren’s syndrome [23,24]. Act1 is a key adaptor protein in IL-17R signaling, as well as a negative regulator of BAFF-BAFFR and CD40-CD40L signaling in B cells [23,24,25,26]. The *Act1-/-* phenotype is associated with increased Th17 cells and Th17-related cytokines (IL-17A, IL-21, IL-22), expanded peripheral B cell populations, hypergammaglobulinemia and autoantibody production, as well as splenomegaly, lymphadenopathy, and mild nephritis. Given the known inhibitory activity of vitamin D3 on both Th17 cells and B cell differentiation and immunoglobulin production, we hypothesized that the absence of dietary vitamin D3 would promote disease development while vitamin D3 supplementation would suppress disease development. To test this hypothesis, we assessed whether 9 weeks of vitamin D3 restriction or supplementation was sufficient to alter the *Act1-/-* autoimmune phenotype; specifically, the development of SLE-like characteristics. We found that dietary vitamin D3 restriction was associated with increased autoantibody and immunoglobulin production, as well as increased percentages of splenic memory B cells, while vitamin D3 supplementation had no significant effect on autoantibody levels and B cell differentiation patterns. Further studies in SLE patients confirmed a negative correlation between levels of memory B cells and vitamin D3, supporting the pathogenicity of vitamin D3 deficiency.

## 2. Materials and Methods

### 2.1. Patient Enrollment

SLE patients seen by a rheumatologist (H.S.) between July 2017 and June 2018 at the Cleveland Clinic Department of Rheumatologic and Immunologic Disease (ages 18–80) were invited to volunteer for an ongoing Lupus Registry. Patients were eligible for inclusion if ACR criteria were met. There were no exclusions based on disease activity, flares, or type of therapy. Demographic information, medical history, and relevant clinical data were collected and managed using REDCap electronic data capture tools hosted at the Cleveland Clinic [27]. At this visit, patients provided blood samples that were processed for serum and peripheral blood mononuclear cells (PBMCs) and frozen at −80 °C, until later processing of all samples, concurrently. Fourteen random patient samples were selected for PBMC B cell analysis as described below. All samples were obtained after patients provided written informed consent and after approval of the study by the Cleveland Clinic Institutional Review Board.

### 2.2. Vitamin D3 Dietary Intervention and Animal Care

*Act1-/-* mice on the Balb/c background were bred at the University of North Carolina Gnotobiotic center, and transferred to specific pathogen-free housing at Lerner Research Institute at 5–7 weeks of age. All mice were born within 3 weeks of each other. Immediately upon arrival at the Lerner Research Institute, the mice were divided into 3 dietary treatment groups—0 IU/kg (low), 2 IU/kg (normal), or 10 IU/kg (high) of vitamin D3/kg chow. The three treatment groups were matched for age and sex to limit potential biases. Mice (*n* = 15) were maintained on their assigned diet for the duration of this 9-week study. Mice were bled for serum at 0, 3, 6, and 9 weeks post-transfer. Due to the immunodeficiency status of *Act1-/-* mice, cages were changed twice weekly and Vetropolycin gel was applied as needed throughout the experiment. Mice had access to hydrogel to prevent dehydration if necessary. All animal procedures were approved by the Cleveland Clinic Institutional Animal Care and Use Committee.

### 2.3. Organ Harvest/Preparation

Tissue samples were both frozen in OCT™ and prepared for paraffin embedding by 24 h fixation in 10% formalin, followed by 80% ethanol. Spleen, submaxillary gland, and cervical lymph nodes were weighed prior to preservation. Kidneys were cut in half longitudinally prior to preservation. Paraffin embedding, sectioning (5 µm), and hematoxylin and eosin staining were performed by the Lerner Research Institute Histology Core. Periodic Acid Schiff (PAS) staining was performed with the Richard–Allan scientific PAS stain kit (Thermo Scientific, Waltham, MA, USA).

Histology measurements were performed on H&E and PAS-stained paraffin-embedded kidney sections. To quantify the mean glomerular area, 8–15 glomeruli per mouse were traced and measured for area using the Keyence BZ-X700 All-in-one microscope, then averaged for each mouse. A renal pathologist (J.N.), blinded to the treatment groups, scored H&E and PAS-stained kidneys for the presence of endocapillary hypercellularity, extracapillary proliferation, immune deposits, tubular atrophy, tubular casts, tubular dilation, and interstitial fibrosis and inflammation. A scale of 0–5 was used for each feature analyzed (8), in which 0 is absent, 1 is 1–5%, 2 is 6–13%, 3 is 11–20%, 4 is 21–50%, and 5 is greater than 50% of the glomeruli/tubules/area of interest, summing to a maximum possible score of 40.

### 2.4. Immunofluorescence Staining of Kidney Tissue

Half kidneys were immediately frozen in OCT™ and subsequently sectioned at 5 µm. Following a brief acetone fixation, sections were stained with anti-IgG or anti-IgM-TexasRed (Southern Biotech, Birmingham, AL, USA) and anti-C’3-FITC antibodies (Immunology Consultants Laboratory, Inc, Portland, OR, USA). Images were obtained using the Keyence BZ-X700 Series at a fixed fluorescence intensity across slides, which was selected to minimize background fluorescence. Mean integrated fluorescence area for IgG- or IgM-TexasRed was calculated per glomerulus and averaged for each mouse. A minimum of 5 glomeruli was evaluated per mouse.

### 2.5. Serum Cytokine Flow Cytometry

Serum was obtained after 9 weeks of treatment and assessed for selected cytokines by a Cytokine Bead Array (BD Bioscience). Briefly, four Flex Sets containing fluorescent beads with pre-conjugated antibodies to each cytokine of interest (IL-1α, IL-6, IL-10, IL-17A) and a fluorescent detection agent were combined and incubated with 50 µl of serum according to the manufacturer’s protocol. Beads with bound cytokines were run on an LSR flow cytometer (BD Biosciences, San Jose, CA, USA) with the assistance of the Lerner Research Institute Flow Cytometry Core. Data were analyzed using FlowJo v10 software.

### 2.6. Enzyme-Linked Immunosorbent Assay (ELISA)

Serum from mice treated for 3–9 weeks was used for all ELISA experiments. Mouse 1,25(OH)_2_D3 and 25(OH)D3 levels were measured by ELISA (MyBioSource and Eagle Bioscience, USA, respectively). Anti-dsDNA IgG and anti-SSB IgG ELISAs were run according to the manufacturer’s guidelines (Alpha Diagnostic International, San Antonio, TX, USA). Immunoglobulin ELISAs were performed as described previously [28]. Briefly, microtiter plates were coated with 2.5µg/mL of unlabeled goat anti-mouse Ig (Southern Biotech) in PBS and blocked with PBS-gelatin. Mouse serum was diluted in assay buffer (5 mg/mL bovine γ-globulin, 5% gelatin, 0.05% Tween-20 in PBS) as follows: IgG (1:200,000), IgG1 (1:100,000), IgG2a (1:100,000), IgG2b (1:200,000), IgG3 (1:75,000), IgA (1:50,000), IgE (1:200,000), IgM (1:50,000). Samples were added to plates and incubated at room temperature for 90 min, then washed with PBS; secondary HRP-conjugated goat anti-mouse antibodies specific for IgG, IgG1, IgG2a, IgG2b, IgG3, IgA, IgE, IgM (all Southern Biotech) were added, followed by incubation for 1 h. Assays were visualized using the TMB substrate kit (Thermo Scientific, Waltham, MA, USA) and read on a Victor 3 plate reader (Perkin Elmer, Waltham, MA, USA) at 450 nm.

### 2.7. Flow Cytometry

At the time of sacrifice (9 weeks of treatment), spleens were isolated. Approximately 50% of each spleen was mashed and red blood cells lysed in ACK buffer (0.15 M NH4Cl, 0.01 M KHCO3, 0.2 mM EDTA) for 5 min. Cells were washed and resuspended in PBS for surface staining against CD3 (145-2C11, PE-Cy7), CD4 (GK1.5, APC), CD8 (53-6.7, PerCP-Cy5.5), B220 (RA3-6B2, PerCP-Cy5.5), CD93/AA4.1 (AA4.1, PE), CD38 (90, FITC), GL7 (GL-7, Biotin), Streptavidin (PE), and/or CD138 (281-2, PE) (all antibodies from eBioscience, Waltham, MA, USA) for 30 min. Samples stained only for surface markers were fixed in 1% paraformaldehyde before analysis. A subset of splenocytes was analyzed for cytokine production after subsequent stimulation with PMA (2 μg/mL) and ionomycin (1 μg/mL) for 4 h at 37 °C, 5% CO_2_, the last 2 h in the presence of 0.4 μL/100 μL cells GolgistopTM (BD Bioscience). Intracellular staining was performed with fluorescence-conjugated antibodies against RORγt, IL-17A, IL-21, and IL-22 (all from eBioscience). All intracellular stains were performed using the Foxp3 transcription factor staining buffer kit (eBioscience) and run within 15 h of permeabilization on the BD LSR Fortessa (BD Biosciences, CA, USA).

Human PBMCs were isolated from SLE patients by Ficoll gradient and stored at −80 °C. On the day of analysis, PBMCs were thawed, washed and resuspended in human Fc Block (BD Bioscience), followed by surface staining against CD19 (HIB19, FITC), CD27 (0323, APC), CD38 (HIT2, Pe-Cy7), and IgD (IA6-2, PE) (all antibodies from eBioscience) in PBS for 30 min. PBMCs were fixed in 1% paraformaldehyde before being run on the BD LSR Fortessa. All data from human and mouse experiments were analyzed using Flowjo v10 software.

### 2.8. Real-Time Reverse-Transcriptase PCR

Splenocytes were isolated from whole spleen as described above and immediately frozen at −80 °C. RNA was isolated using the RNAeasy Plus Micro Kit (Qiagen, Valencia, CA) and converted into cDNA using the qScript cDNA SuperMix (Quanta BioSciences, Gaithersburg, MD, USA). PCR was subsequently performed using 100 ng cDNA and the PerfeCTa^®^ SYBR^®^ Green FastMix^®^ ROX (Quanta BioSciences) in the 7300 Real-Time PCR System (Applied Biosystems, Foster City, CA, USA). β-actin was used as the internal control for all RT-PCR experiments. All primers were created by Integrated DNA Technologies (Skokie, IL). PCR analyses were performed in triplicate, or duplicate for β-actin. Primer sequences were as follows: *Aicda* 5′-GCGGACATTTTTGAAATGGTA-3′ and 3′-GGGAGTTTCAGAATCCGGTT-5′; *Rgs13* 5′-ATCTACATCCAGCCACAGTCTC-3′ and 3′- TCAGGAGTTGTTGGTACATTTCAG-5′; *Il6ra* 5′-ACACACTGGTTCTGAGGGAC-3′ and 3′-GTGGACGGTTGGAACACCAT-5′; *Itga4* 5′-TGTGCAAATGTACACTCTCTTCCA-3′ and 3′-CTCCCTCAAGATGATAAGTTGTTCAA-5′; *Actb* 5′-TGGGAATGGGTCAGAAGGAC-3′ and 3′-GGGTCTAGTACAAACTCTGG-5′.

### 2.9. Statistical Analyses

Comparisons between treatment groups were analyzed by Student’s *t*-test with Welch’s correction for non-normally distributed samples. Two-way ANOVA was used to test for statistical differences in vitamin D3 levels over time in study animals. Associations between vitamin D3 and PBMC isolated cells were analyzed by linear regression, with reported r^2^ values after checking for linearity and error of variance. Statistical analyses and graphs were performed in GraphPad Prism (La Jolla, CA, USA) or JMP Pro version 14 (SAS Institute, Cary, NC, USA). Significance level was set at alpha = 0.05 for all analyses.

## 3. Results

### 3.1. Serum 25(OH)D3 Levels Plateau after Controlled Dietary Vitamin D3 Intake

**Figure 1:**
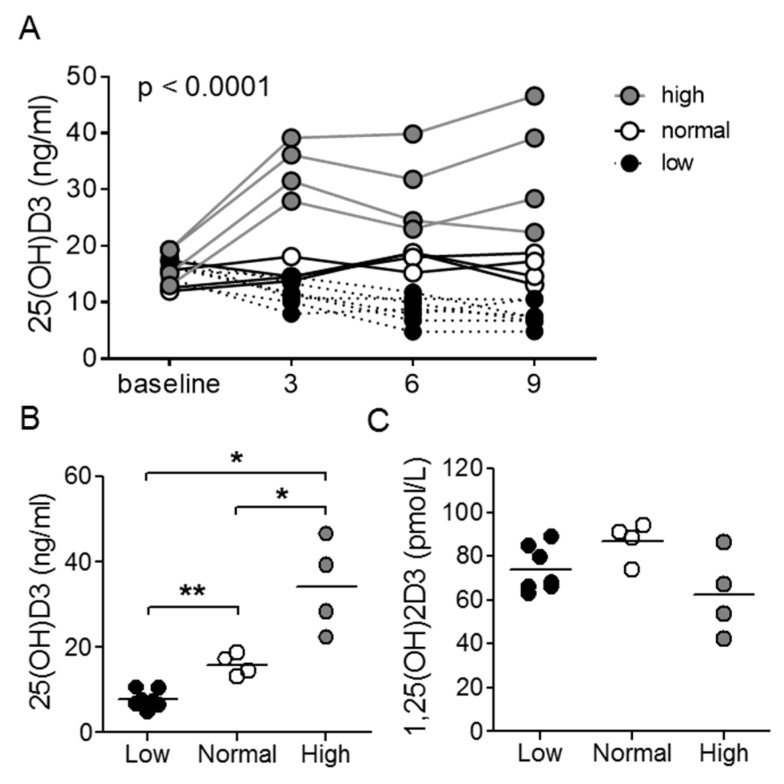
25(OH)D3 chow alters serum 25(OH)D3, but not 1,25(OH)_2_D3 levels. (**A**) Longitudinal and (**B**) 9-week assessment of serum 25(OH)D3 levels in *Act1-/-* mice. (**C**) 9-week analysis of serum 1,25(OH)_2_D3 levels in *Act1-/-* mice. In (**A**), each line represents a single mouse, and *p*-value reflects two-way ANOVA, and in (**B**,**C**), each symbol represents an individual mouse with a horizontal line representing the group mean. * *p* < 0.05, ** *p* < 0.01; unpaired *t*-test with Welch’s correction.

Act1-deficient mice develop a lupus-like disease characterized by hyper-Th17 differentiation and B cell hyperplasia. Using this model, we analyzed the immunoregulatory effect of vitamin D3. It was previously reported that serum 25(OH)D3 levels plateau after 5 weeks of dietary intervention [29]. Therefore, to ensure stabilization of vitamin D3 levels, all mice underwent 9 weeks of treatment with low (0 IU/g chow), normal (2 IU/g chow), or high (10 IU/g chow) vitamin D3 diets prior to analysis. We determined levels of serum 25(OH)D3 as an indicator of vitamin D3 status, as the hormonally active 1,25(OH)_2_D3 is tightly regulated with a half-life in the order of hours [30,31]. Serum 25(OH)D3 levels significantly differed between treatment groups already after 3 weeks and remained stable thereafter (Figure 1A,B). As anticipated, there were no differences in serum 1,25(OH)_2_D3 between the groups of mice (Figure 1C).

### 3.2. Vitamin D3 Manipulation Does Not Affect Th17 Cells in the Act1-/- Mouse

**Figure 2:**
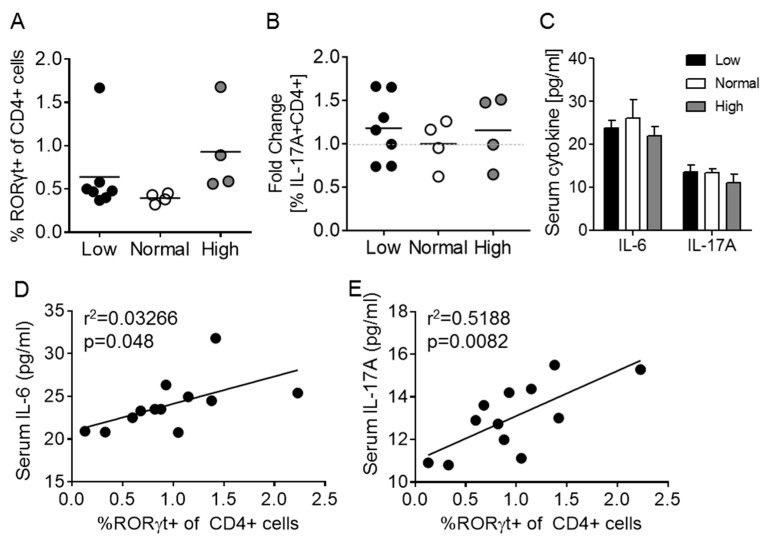
Dietary vitamin D3 manipulation does not affect Th17 cells in the *Act1-/-* mouse. (**A**) Splenic Th17 cells (CD3+CD4+RORγt+) and (**B**) IL-17A production by splenic Th17 cells stimulated with PMA and ionomycin. (**C**) IL-1α, IL-6, and IL-17A cytokine profile after 9 weeks of dietary vitamin D3. (**D**,**E**) Association between splenic circulating Th17 cells and serum levels of IL-6 (**D**) and IL-17A (**E**). Each symbol represents an individual mouse. In (**A**,**B**), the mean of each group is represented by a horizontal line, and in (**D**,**E**) a best fit line is shown.

Th17 cells were identified as key contributors to the *Act1-/-* phenotype [32,33,34]. Furthermore, it was shown that inhibition of Th17-related cytokines (IL-17A, IL-21, IL-22) prevented specific autoimmune features [32,33]. Since vitamin D3 has been shown to reduce Th17 differentiation in vitro and in vivo [14,35,36,37], vitamin D3 was proposed as a potential therapy for the *Act1-/-* mouse. Contrary to previous studies, dietary differences in vitamin D3 did not alter the Th17 cell population (CD4+RORγt+) in the *Act1-/-* mouse model (Figure 2A; see Supplemental Figure S1 for gating strategy). Additionally, PMA/ionomycin-stimulated *Act1-/-* splenocytes demonstrated comparable IL-17A production between vitamin D3 groups (Figure 2B), which corresponded with no difference in serum IL-17A between treatment groups (Figure 2C). This was further supported by a lack of difference in serum IL-6 (critical for Th17 differentiation) levels between treatment groups (Figure 2C). It should be noted that despite a lack of association with vitamin D3 levels, a positive correlation between numbers of circulating Th17 cells and serum levels of IL-6 and IL-17A was observed as expected (Figure 2D,E).

### 3.3. Vitamin D3 Restriction Specifically Augments Memory B Cells

**Figure 3:**
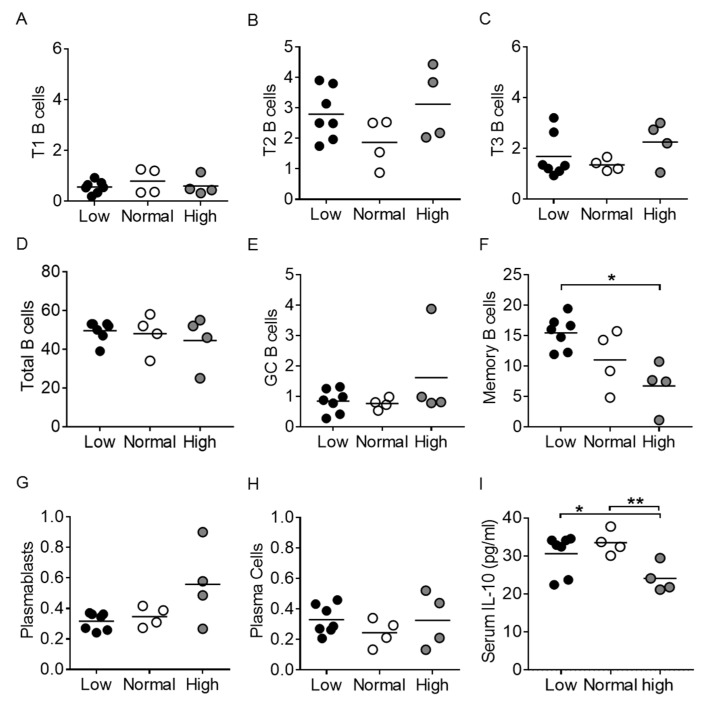
Memory B cells are specifically affected by vitamin D3 restriction. Splenocytes were analyzed for populations of (**D**) total B220+ B cells, (**A**–**C**) transitional B cells—T1 (B220+AA4.1+IgM+CD23-), T2 B220+AA4.1+IgM+CD23+), and T3 (B220+AA4.1+IgM-CD23+), (**E**) germinal center B cells (B220+IgM-GL7+CD38^low^), (**F**) memory B cells (B220+IgM-CD38^hi^GL7-), (**G**) plasmablasts (B220-CD138+IgM+) and (**H**) plasma cells (B220-CD138+IgM-). (**I**) Serum IL-10 levels. Each symbol represents an individual mouse, and the mean of each group is represented by a horizontal line. * *p* < 0.05, ** *p* < 0.01; unpaired *t*-test with Welch’s correction. GC = germinal center.

It was previously shown that the *Act1-/-* mouse displays increased numbers of peripheral B cells and plasma cells due to Act1′s role as a negative regulator of B cell survival and expansion [23,25,26]. Furthermore, it is well-established that *Act1-/-* mice develop elevated levels of serum autoantibodies [23,25,38]. Splenic B cell populations were analyzed to assess the role of vitamin D3 in peripheral B cell populations and autoantibody production (see Supplemental Figure S2 for gating strategy). We found no differences in total B220+ B cells, as well as no differences in transitional T1 (B220+AA4.1+IgM+CD23-; *p* = 0.71), T2 (B220+AA4.1+IgM+CD23+; *p* = 0.38), or T3 (B220+AA4.1+IgM-CD23+; *p* = 0.22) B cell subsets between treatment groups (Figure 3A–D). Germinal center (GC) B cells (B220+IgM-GL7+CD38low) were also similar across vitamin D3 groups (low vs. normal, *p* = 0.66; low vs. high, *p* = 0.38; normal vs. high, *p* = 0.34) (Figure 3E,F); however, the vitamin D3-restricted group displayed significantly higher levels of memory B cells (B220+IgM-CD38hiGL7-) compared to the high vitamin D3 group (*p* < 0.05) (Figure 3C). Surprisingly, the percentage of plasmablasts (B220-CD138+IgM+) remained unaffected by vitamin D3 manipulation (low vs. normal, *p* = 0.46; low vs. high, *p* = 0.16; normal vs. high, *p* = 0.21) as did plasma cells (B220-CD138+IgM-; low vs. normal, *p* = 0.19; low vs. high, *p* = 0.97; normal vs. high, *p* = 0.47) (Figure 3G,H). Thus, vitamin D3 treatment selectively impaired memory B cells in *Act1-/-* mice.

In a gene array study of naïve, germinal center, memory, and plasma cells, *Rgs13* expression was identified as specific to germinal center B cells, whereas *Itga4* and *Il6ra* gene expression was specific for memory B cells [39]. It should be noted though that *Il6ra* transcripts are also widely present in T cells and myeloid cells [40,41], and while *Aicda* gene expression is commonly used as a marker for the germinal center B cell as AID (activation-induced deaminase) is critical for class-switching and somatic hypermutation, *Aicda* is also expressed in memory B cells, albeit at a lower level [39]. Finally, *Bcl6* is expressed highly by memory B cells, but also in T follicular helper cells and memory T cells [42,43]. We found no difference in *Rgs13* gene expression levels from whole spleen from vitamin D3-manipulated *Act1-/-* mice, supporting a lack of difference in levels of GC B cells (Table 1). In contrast, *Aicda* and *Bcl6* gene expression was significantly reduced in the high vitamin D3 group (Table 1), potentially as a result of the reduced levels of memory B cells (see Figure 3C). *Itga4* gene expression levels were similarly reduced (*p* = 0.054) in the high vitamin D3 group, further supporting a reduction in memory B cells. We found no differences in *Il6ra* gene expression in these total spleen samples. Taken together, gene transcript levels support a specific effect of vitamin D3 on memory B cell differentiation in *Act1-/-* mice.

IL-10 is a pleiotropic cytokine with both immunostimulatory and immunoregulatory roles [44]. IL-10 has been found to be involved in the germinal center reaction and as a driver of differentiation of naïve B cells into memory B cells and plasma cells [45]. We analyzed vitamin D3-treated *Act1-/-* mice for serum IL-10 levels and found significantly increased levels in the low and normal vitamin D3 groups as compared with the high group (*p* < 0.05–0.01) (Figure 3I).

### 3.4. Elevated Immunoglobulin Production and Anti-dsDNA in Vitamin D3-Restricted Mice

**Figure 4:**
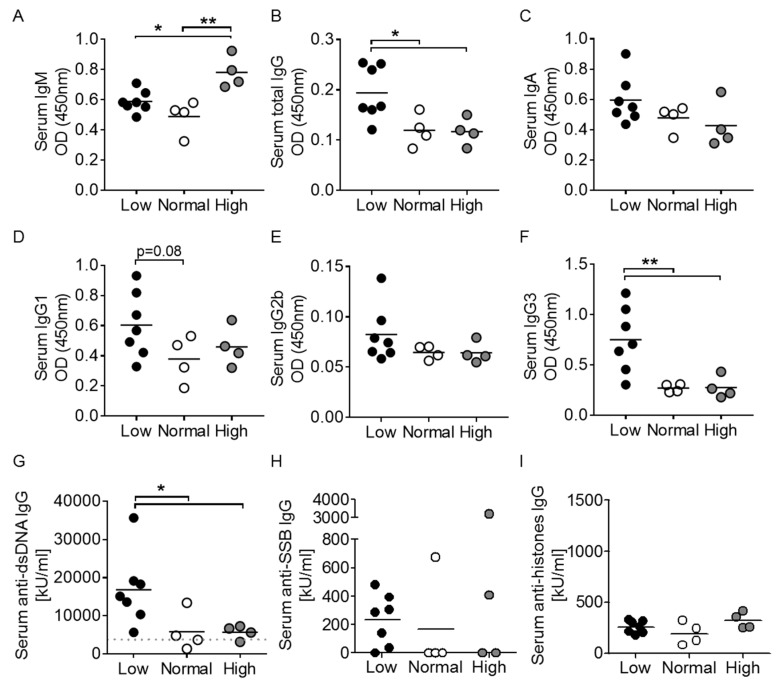
Immunoglobulin and autoantibody production are affected by vitamin D3 manipulation in *Act1-/-* mice. (**A**–**F**) Serum immunoglobulins and (**G**–**I**) serum autoantibodies (anti-dsDNA IgG, anti-SSB IgG, and anti-histone IgG) were measured by ELISA. Each symbol represents one individual mouse, and the mean of each group is represented by a horizontal line. * *p* < 0.05, ** *p* < 0.01; unpaired *t*-test with Welch’s correction.

The *Act1-/-* mouse displays hypergammaglobulinemia, as well as elevations in specific immunoglobulin subtypes including IgG1, IgG2a, IgG2b, and IgE [23]. After 9 weeks of vitamin D3 manipulation, serum IgM levels were significantly greater in the high vitamin D3 group compared to both the low and normal vitamin D3 groups (Figure 4A). Oppositely, total IgG levels were found to be significantly higher in the vitamin D3 low group (Figure 4B), supporting B cell activation in vitamin D3-restricted *Act1-/-* mice. We found no statistical differences in serum IgA, IgE, IgG1, or IgG2b between the groups, although a trend for higher Ig levels in the low vitamin D3 group was identified for IgA, IgG1 and IgG2b (Figure 4C–E and data not shown). Interestingly, there were significantly elevated IgG3 levels in the vitamin D3 low group only (Figure 4F), suggesting a specific effect of vitamin D3 on limiting Ig class switching to IgG3.

Anti-nuclear autoantibodies are classic features of mouse lupus-like disease, and *Act1-/-* mice at 3.5 months were previously shown to express elevated anti-dsDNA IgG autoantibodies and to have detectable, albeit very low levels of anti-SSB IgG autoantibodies [24]. We assessed *Act1-/-* serum for the presence of anti-dsDNA, anti-SSB and anti-histones IgG autoantibodies. Vitamin D3-restricted mice displayed significantly elevated serum levels of anti-dsDNA IgG autoantibodies (Figure 4G), while no differences were detected in anti-SSB and anti-histones IgG levels between the groups (Figure 4H,I). Despite a lack of significance, it should be noted that 6/7 mice in the low vitamin D3 group displayed detectable levels of anti-SSB, compared to only 1/4 and 2/4 mice in the normal and high vitamin D3 groups, respectively.

### 3.5. Vitamin D3 Exposure Does Not Affect Features of Nephritis after 9 Weeks

**Figure 5:**
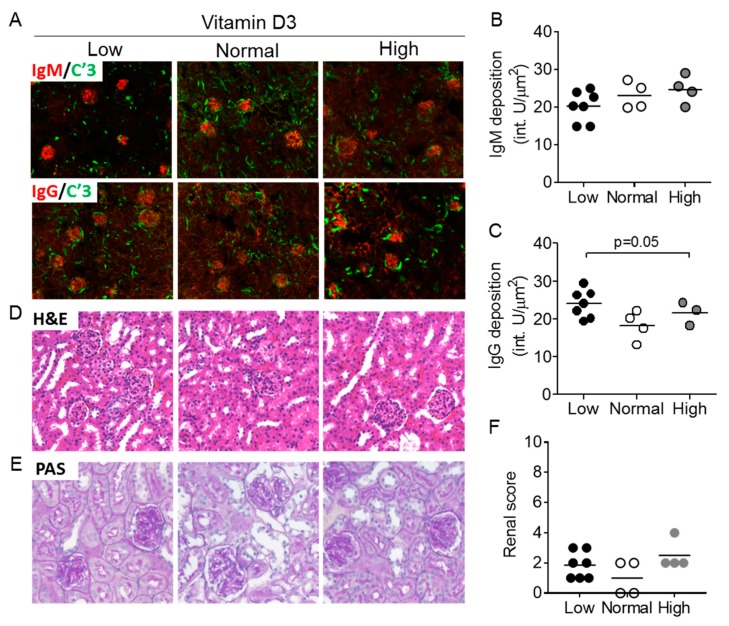
Renal immunoglobulin deposition and inflammation is unaffected by vitamin D3 manipulation in *Act1-/-* mice. (**A**) *Act1-/-* kidneys were stained for IgM (red) and C’3 (green) or IgG (red) and C’3 (green), and analyzed for (**B**) IgM and (**C**) IgG deposition in the glomeruli. Kidneys were also stained by (**D**) H&E and (**E**) PAS to assess glomerular and tubular inflammation and injury which is combined into a renal score (**F**). Each symbol represents an individual mouse and the mean of each group is represented by a horizontal line. * *p* < 0.05, ** *p* < 0.01; unpaired t-test with Welch’s correction.

Glomerulonephritis in the *Act1-/-* mouse is typically mild, characterized by IgG and IgM glomerular deposition at 8–12 months [24]. Analysis of Ig deposition in the kidneys showed similar levels of IgM-immune complexes in the glomeruli between the treatment groups (Figure 5A,B). We observed increased IgG deposition in the low vitamin D3 group compared to the high vitamin D3 group, which corresponded with total IgG/IgG3 and anti-dsDNA IgG patterns in *Act1-/-* sera (*p* = 0.05) (Figure 5C). Quantification of renal histological findings from H&E and PAS-stained kidneys showed no difference between groups (Figure 5D–F).

### 3.6. Memory B Cells Are Negatively Associated with Vitamin D3 in SLE

**Figure 6:**
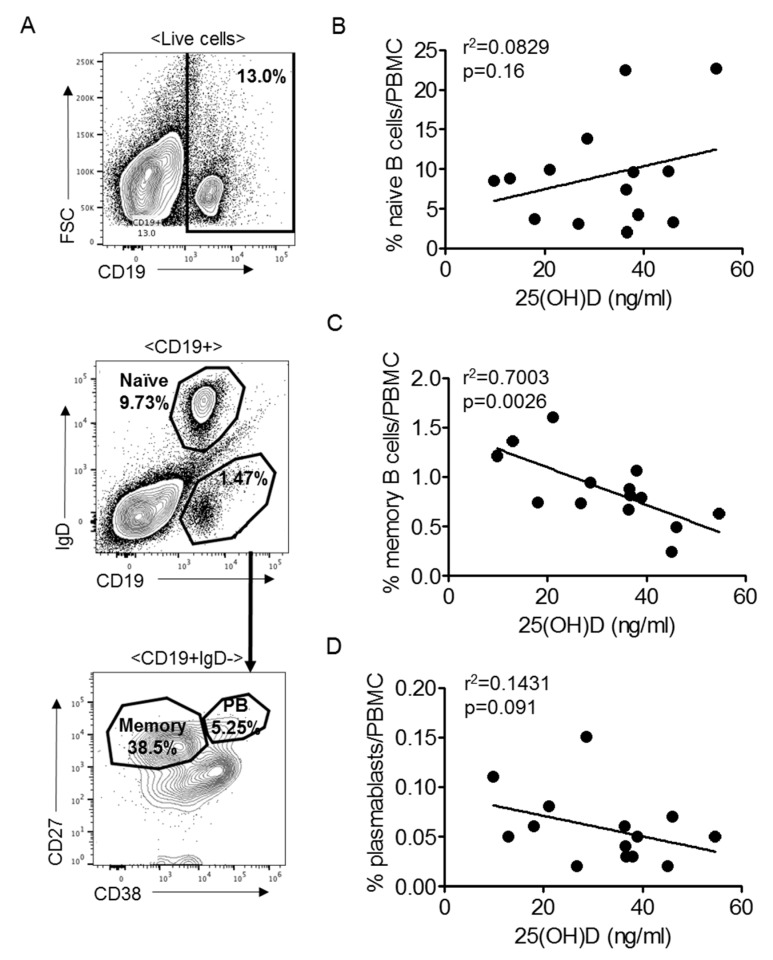
Memory B cells are negatively correlated with vitamin D levels in SLE patients. (**A**) Gating strategy for Figure 25(OH)D levels against circulating (**B**) naïve B cells (CD19+IgD+), (**C**) memory B cells (CD19+IgD-CD27+CD38-) and (**D**) plasmablasts (CD19+IgD-CD27+CD38+) displayed as percent of live cells. Each symbol represents an individual patient, *n* = 14. PB = plasmablast.

To determine if the specific difference in memory B cell levels observed in *Act1-/-* mice was also detectable in human SLE patients, we tested PBMC samples from 14 SLE patients (Table 2). All patients were well-controlled, with SLEDAI-2K clinical severity scores ranging from 0 to 8. Vitamin D3 levels ranged from 9.9 to 54.7 ng/mL, with 46% of patients presenting as vitamin D3 deficient (<30 ng/mL as per institutional laboratory standards). Corresponding with data from the *Act1-/-* mouse, SLE patient serum 25(OH)D3 was negatively correlated with memory B cells (CD19+IgD-CD27+CD38-) (*p* < 0.01) (Figure 6A,C), while there was no correlation between serum 25(OH)D3 and levels of naïve B cells (CD19+IgD+) (*p* = 0.16) or plasmablasts (CD19+IgD-CD27+CD38+) (*p* = 0.091) (Figure 6B,D).

## 4. Discussion

Persistent in the autoimmune literature is the lack of clarity regarding the role of vitamin D3 in autoimmune disease. Despite pervasive vitamin D deficiency across autoimmune diseases, there has been little consensus on a mechanism explaining how vitamin D deficiency relates to disease features, and few studies have explored these mechanisms in animal models of SLE [6,7,8,9,46]. In this study, we explored the relationship between low, normal and high vitamin D3 exposure and lupus-like disease in the *Act1-/-* mouse and in a cohort of well-controlled SLE patients. Interestingly, we found that low vitamin D3 levels specifically affected memory B cells and, at least in the mouse model, antibody production.

Spontaneous autoimmune models often require time to develop features of autoimmunity. Despite a short experimental timeframe of 9 weeks, we detected significant elevations in anti-dsDNA IgG antibody levels and total immunoglobulin levels (total IgG and IgG3) in mice fed a vitamin D3-restricted diet compared to mice fed diets containing normal or high vitamin D3. Levels of IgA, IgG1 and IgG2b did not reach statistical significance, but nevertheless trended in a similar fashion, while serum IgM levels showed a reciprocal pattern. Interestingly, we observed a significant decrease in serum IL-10 in the high vitamin D3 group, and IL-10 has been shown to promote IgG1 and IgG3 production by human B cells [47]. Higher levels of IL-10 in the low and normal vitamin D3 groups might therefore contribute to the observed elevations in IgG1 and IgG3. If so, it is interesting that vitamin D3 treatment in this mouse model led to a reduction in IL-10, thus suggesting that IL-10 plays a pathogenic rather than anti-inflammatory role in the *Act1-/-* mouse. A similar relationship has been reported in both mouse models of lupus and SLE patients [48,49,50,51,52]. Additionally, the low number of mice in each of the treatment groups must be considered when interpreting these data.

Anti-dsDNA IgG is a strong marker of lupus-like disease and was highly elevated in *Act1-/-* mice fed a low vitamin D3 diet. However, no differences were observed between the normal and high vitamin D3 groups. This finding suggests that a lack of vitamin D3 has a greater impact on disease than high vitamin D3 supplementation. In fact, the majority of our data suggest that high amounts of vitamin D3 fail to provide additional benefit compared to an adequate amount of vitamin D3. This observation is somewhat contradictory to the available data from vitamin D3-manipulated MRL-lpr/lpr lupus-prone mice [6,7,8]. One explanation may be that these studies tested the efficacy of active vitamin D3 analogs (i.e., 1,25(OH)_2_D3, 1,24R(OH)_2_D3, 22-oxa-1,25(OH)_2_D3), as an intervention, while our study was aimed at evaluating the effect of 25(OH)D3, as this is the form frequently used in clinical studies [53]. Although vitamin D3 levels were significantly different and stable already after 3 weeks of treatment, providing the animals with steady-state levels of vitamin D3 for six weeks, it is also possible that constitutive high vitamin D3 levels are required beyond the limited disease course of 9 weeks. As such, a longer interventional period might have also elicited measurable differences between the normal and high vitamin D3 groups. The fact that the *Act1-/-* mice studied here did not display the overt splenomegaly or lymphadenopathy commonly seen in *Act1-/-* mice [23,24] also supports this explanation (data not shown).

The *Act1-/-* mouse has an intrinsic drive for Th17 development due to unregulated STAT3 activity leading to Th17 differentiation [34]. Despite support in the literature for a role for vitamin D3 in reducing Th17 cell differentiation [14,54,55], we did not observe any changes in the splenic Th17 cell population, serum IL-17A, or stimulated production of IL-17A. It is possible that the mechanism of vitamin D3-mediated inhibition of Th17 differentiation occurs upstream of STAT3, which might explain why no differences in Th17 cells were observed via staining for RORγt. Alternatively, the intrinsic drive towards Th17 development might have a greater influence on Th17 differentiation than vitamin D3. Further studies are needed to determine the exact target of vitamin D3 in Th17 differentiation and function.

Given the correlation between autoantibody titer and SLE disease activity, it is reasonable to infer that vitamin D3′s association with SLE may be attributable to its role in the regulation of splenic B cell differentiation. In fact, we found the B cell compartment to be the primary target of vitamin D3 restriction in the *Act1-/-* mouse. Vitamin D3 restriction specifically promoted memory B cells, without affecting germinal center B cells or plasmablasts/plasma cells. Increased *Aicda, Bcl6*, and *Itga4* expression in the low vitamin D3 group further supported these data. Consistent with these data, vitamin D3 supplementation demonstrated transcriptional regulation of VDR in the germinal center and inhibition of *Aicda* expression (encoding AID, activation-induced deaminase), a cytidine deaminase critical for class-switch recombination and somatic hypermutation [56]. While expression of *Bcl6* is not limited to B cells, it is interesting to note that the protein is also found in T follicular helper cells and memory T cells [42,43], and thus reduced expression corresponds with reduced humoral immune activation in general. The relationship between memory B cells and vitamin D3 was further supported by a negative correlation between serum 25(OH)D3 and memory B cells, but not total B cells and plasmablasts, in PBMC samples from SLE patients. Overall, these observations are consistent with early studies on B cell development that showed inhibition of B cell activation and differentiation by vitamin D3 metabolites and analogs [17,20,21,22].

It is puzzling that the observed difference in memory B cells is not mimicked in the populations of plasmablasts and plasma cells, as would be expected if the memory B cells were a result of T cell-dependent germinal center activity. T cell-independent memory B cells (B1b cells) have previously been identified as a population of B cells primarily located in the peritoneal and pleural cavities with the capacity to develop memory phenotypes and produce immunoglobulins without T cell interactions, thereby bypassing the germinal center [57]. Therefore, the isolated effect observed on memory B cells could be a consequence of the development of T cell-independent memory B cells. This possibility is further supported by the high level of IgG3 identified in the low vitamin D3 group, as it has been shown that in particular IgG3 is produced in response to T cell-independent type 2 antigens [58,59]. Unfortunately, there are surprisingly few data in the clinical literature regarding memory B cells and vitamin D3 levels in SLE patients, though evidence from multiple sclerosis (MS) studies has shown an association between lower intrathecal 25(OH)D3 and increased class-switched memory B cell accumulation in the CSF during MS relapses [19].

Finally, the origin of vitamin D deficiency in SLE is unknown. It is possible that the vitamin D deficiency observed in SLE patients is a result of reduced sun exposure due to photosensitivity; however, such a correlation remains speculative. Regardless of the cause, vitamin D deficiency is widely believed to play a role in pathogenesis. In support thereof, the few animal studies available have demonstrated improvement in lupus features when supplemented with 1,25(OH)_2_D3 [6,7,60], yet no improvement was observed when animals were supplemented with 25(OH)D3 [46]. Based on these studies, it is possible that calcitriol supplementation is needed to achieve clinical improvement. Such a requirement may explain the lack of clinical success, as calcitriol, but not 25(OH)D3, supplementation carries a risk of toxicity and is therefore not typically used for SLE patients without severe kidney disease. Investigations into whether there are therapeutic differences between the two and whether 1,25(OH)_2_D3 can be therapeutic at non-toxic concentrations will be interesting.

## 5. Conclusions

In conclusion, vitamin D3 deficiency has been established as a risk factor for lupus, yet more studies focus on the effect of supplementation rather than the effect of deficiency. The majority of the studies on vitamin D3 in mouse models of lupus compare normal vitamin D3 exposure to high levels of 1,25(OH)_2_D3, the hormonally active form of vitamin D3, while clinically, patients are supplemented with cholecalciferol (inactive vitamin D3), due to the increased risk of hypercalcemia in response to 1,25(OH)_2_D3 as compared to cholecalciferol or 25(OH)D3 [61]. Our study is unique in that it compared the effect of a low vitamin D3 diet with that of both normal and supplemented (high) vitamin D3 states using 25(OH)D3, the intervention that is also used for treatment clinically. This approach allowed us to identify memory B cells as a functional result of vitamin D3 restriction in an autoimmune-prone model.

## Figures and Tables

**Table 1:** Whole spleen gene expression.

	Vitamin D3		
Gene	Low	Normal	High
Rgs13	1.2 ± 0.1	1.1 ± 0.1	1.4 ± 0.2
Bcl6	0.8 ± 0.1	1.0 ± 0.1	0.3 ± 0.02 ^ac^
Aicda	2.5 ± 0.5	2.4 ± 0.9	1.1 ± 0.3 ^a^
Itga4	0.8 ± 0.1	1.0 ± 0.1	0.6 ± 0.2 ^b^
Il6ra	1.3 ± 0.3	1.0 ± 0.1	1.4 ± 0.2
Irf4	1.2 ± 0.1	1.0 ± 0.1	0.8 ± 0.2

^a^*p* < 0.05 versus low; ^b^ *p* ≤ 0.05, ^c^ *p* < 0.01 versus normal.

**Table 2:** Systemic Lupus Erythematosus (SLE) patient characteristics.

Pt	Sex	Age	Race	25(OH)D3 (ng/mL)	SLEDAI -2K	anti-dsDNA (IU/mL)	C3/C4	Cr	IL- 17A	Th17 cells	%Naive B cells	%Memory B cells	%Plasma-blasts	Medication
1	F	33	Other	45.1	6	-	114/46	-	14.0	0.4	9.73%	0.24%	0.02%	Hydroxychloroquine, Mycophenolate
2	F	36	White	38.0	6	n.d.	-	-	6.0	0.68	9.58%	1.06%	0.03%	-
3	F	68	Black	46.1	2	102	-	0.81	10.0	0.91	3.27%	0.49%	0.07%	Hydroxychloroquine, Oral steroids
4	F	57	White	36.4	4	n.d.	160/37	0.62	9.5	0.81	22.44%	0.67%	0.06%	Methotrexate, Oral steroids
5	M	70	White	54.7	2	n.d.	-	2.07	7.0	0.89	22.65%	0.63%	0.05%	Hydroxychloroquine
6	F	54	White	38.9	2	n.d.	146/26	0.77	8.0	0.66	4.24%	0.79%	0.05%	Hydroxychloroquine
7	F	36	Other	28.7	6	46	105/14	0.67	64.6	1.42	13.83%	0.94%	0.15%	Hydroxychloroquine, Oral steroids
8	F	39	Black	13.1	6	n.d.	105/18	0.75	32.7	0.8	8.75%	1.36%	0.05%	Hydroxychloroquine, Mycophenolate, Oral steroids
9	F	45	White	18.1	4	n.d.	186/35	0.67	1.8	2.19	3.72%	0.74%	0.06%	Hydroxychloroquine, Oral steroids
10	F	45	White	36.8	4	n.d.	145/44	0.89	23.8	0.76	2.04%	0.81%	0.03%	Hydroxychloroquine, Oral steroids
11	F	28	Black	9.9	2	n.d.	117/23	0.52	2.2	1	8.52%	1.21%	0.11%	-
12	F	39	White	26.8	0	n.d.	145/30	2.63	16.5	1.47	3.10%	0.73%	0.02%	Hydroxychloroquine, Oral steroids
13	F	39	Other	21.1	3	-	65/12	0.7	17.8	0.56	9.87%	1.60%	0.08%	Hydroxychloroquine, Mycophenolate
14	F	61	White	36.6	8	12	121/28	0.83	306.2	0.12	7.41%	0.88%	0.04%	Oral steroids

Th17 cells (CD4+RORγt+) are reported as percentage of CD4+ cells. Naïve B cells, memory B cells and plasmablasts from peripheral blood mononuclear cells (PBMCs) are reported as percentage of live cells. Pt = patient; SLEDAI-2K = Systemic Lupus Erythematosus Disease Activity Index-2000; Cr = creatinine; n.d. = not detectable; - = not done.

## Supplementary Materials

The following are available online at https://www.mdpi.com/2072-6643/12/2/291/s1, Figure S1: Th17 gating strategy, Figure S2: B cell gating strategy.
