# Supplementary material for: Low Levels of Vitamin D Promote Memory B Cells in Lupus
Source: Nutrients. 2020 Jan 22;12(2):291. doi: 10.3390/nu12020291 (PMC7070834; doi:10.3390/nu12020291)
Supplement: Supplementary file 1 [file nutrients-12-00291-s001.pdf]

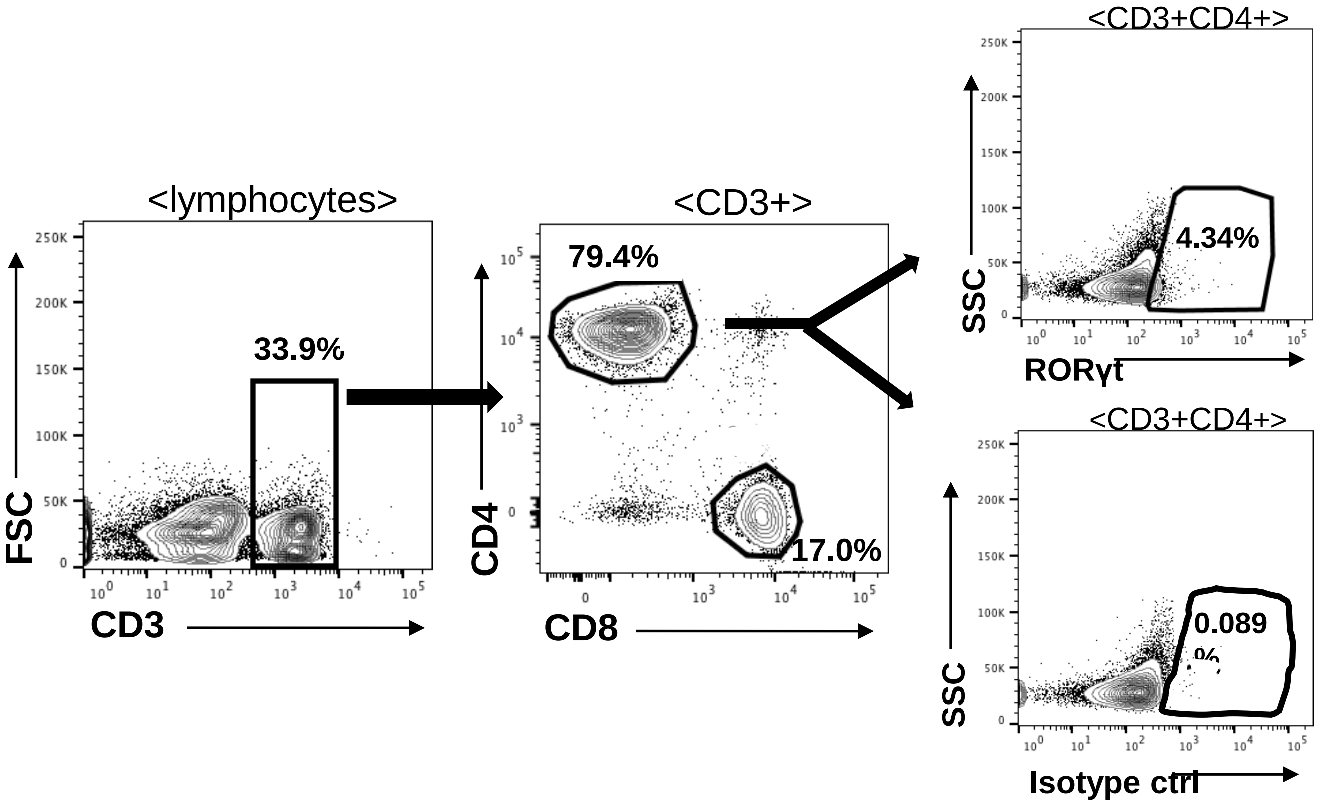

Supplemental Figure 1. Gating strategy for Th17 cells from whole spleen and splenocyte stimulation. Splenocytes were assessed for Th17 cells either with (CD3+CD4+RORγt+) or with (CD3+CD4+IL-17A) stimulation by PMA/ionomycin. Isocontrol antibodies for intracellular staining (RORγt and IL-17A) were used as a negative control.

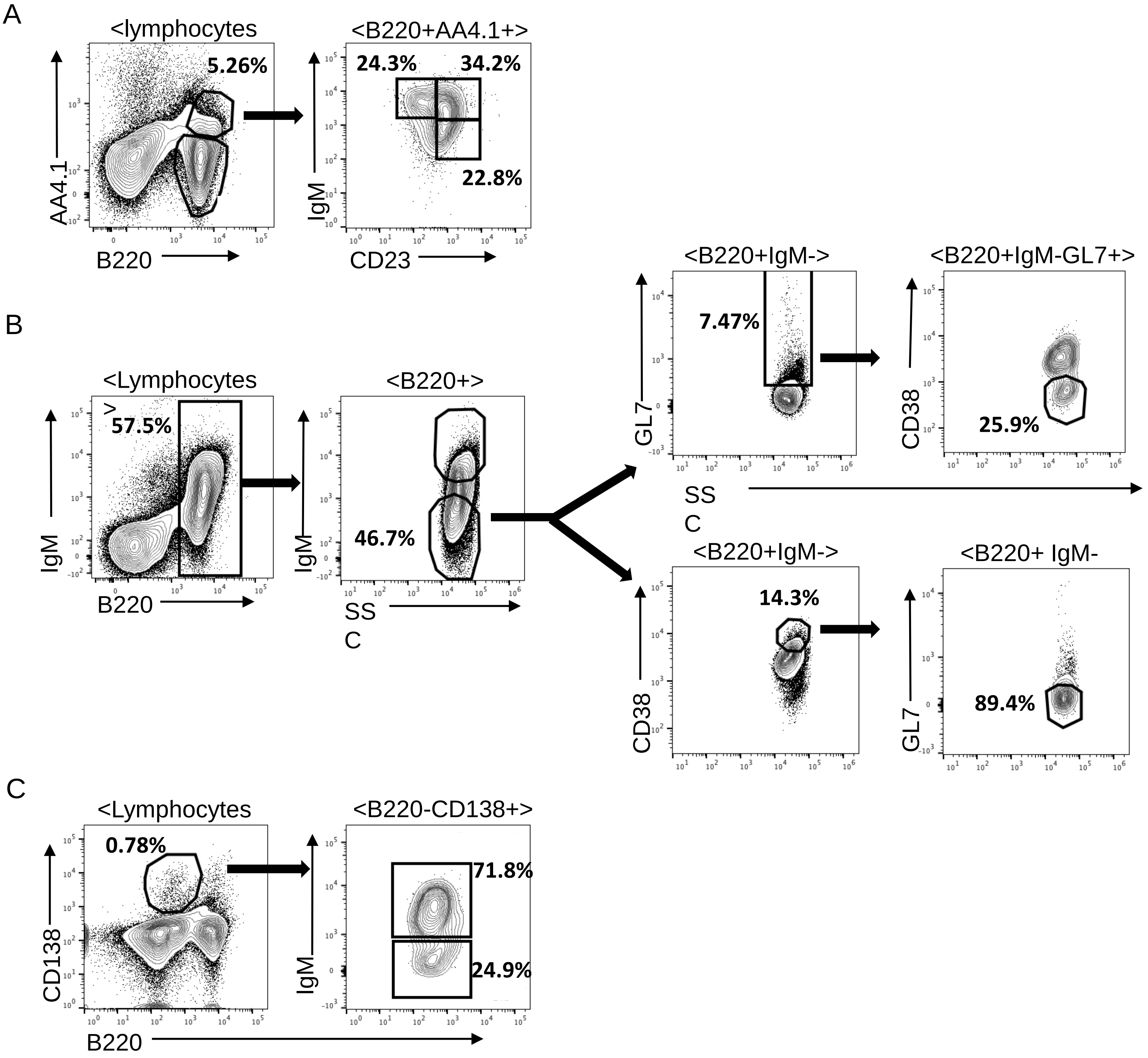

Supplemental Figure 2. Gating strategy for *Act1-/-* B cell flow analysis. A) Transitional B cells, B) Germinal center (B220+IgM-GL7+CD38^{low}, top) and Memory B cells (B220+IgM-CD38^{hi}GL7-, bottom), C) plasmablasts (B220-CD138+IgM+) and plasma cells (B220-CD138+IgM-).
